# Addition of Olive Pomace to Feeding Substrate Affects Growth Performance and Nutritional Value of Mealworm (*Tenebrio Molitor* L.) Larvae

**DOI:** 10.3390/foods9030317

**Published:** 2020-03-10

**Authors:** Sara Ruschioni, Nino Loreto, Roberta Foligni, Cinzia Mannozzi, Nadia Raffaelli, Federica Zamporlini, Marina Pasquini, Andrea Roncolini, Federica Cardinali, Andrea Osimani, Lucia Aquilanti, Nunzio Isidoro, Paola Riolo, Massimo Mozzon

**Affiliations:** Department of Agricultural, Food and Environmental Sciences, Università Politecnica delle Marche, Via Brecce Bianche 10, 60131 Ancona, Italy; s.ruschioni@staff.univpm.it (S.R.); n.loreto@pm.univpm.it (N.L.); r.foligni@staff.univpm.it (R.F.); c.mannozzi@staff.univpm.it (C.M.); n.raffaelli@staff.univpm.it (N.R.); f.zamporlini@staff.univpm.it (F.Z.); m.pasquini@staff.univpm.it (M.P.); a.roncolini@pm.univpm.it (A.R.); f.cardinali@pm.univpm.it (F.C.); a.osimani@staff.univpm.it (A.O.); l.aquilanti@staff.univpm.it (L.A.); n.isidoro@staff.univpm.it (N.I.)

**Keywords:** mealworm, *Tenebrio molitor*, pomace, olive by-products, fatty acids

## Abstract

The well-recognized efficiency of *Tenebrio molitor* larvae to convert low quality organic matter into a nutritionally valuable biomass was exploited to manage solid wastes coming from the olive oil industry, which represent a severe environmental challenge in the Mediterranean area. Three organic pomace-enriched substrates (mixtures middlings/pomace 3:1, 1:1, and 1:3) were assessed, together with 100% organic wheat flour and 100% organic middlings as control feeds. A feeding substrate made up of 25% olive pomace and 75% wheat middlings appeared to be the best compromise between growth performance (larval and pupal weights, survival rate, development time) and nutritional properties of mealworm larvae. In fact, larvae fed the 3:1 feed showed the highest dry matter (DM) yield (38.05%), protein content (47.58% DM), and essential/non-essential amino acids ratio (1.16). Fat content (32.14% DM) and fatty acid composition were not significantly different than those of larvae fed more pomace-enriched feeds.

## 1. Introduction

Olive oil represents a traditional product of vital economic significance for many Mediterranean countries. However, fruits processing generates huge volumes of solid residues, called olive pomace or olive husk, and liquid wastewaters [1]. The high BOD (biochemical oxygen demand) and COD (chemical oxygen demand) values of wastewaters, the presence of phytotoxic materials (high organic acids and polyphenols levels), the strong odor, and the doughty texture of solid wastes affect by-product management and limit their uses “as is”. Therefore, several valorization options have been explored to both reduce the ecological impact and increase the added value of by-products from the olive oil industry, such as recovering nutritionally valuable substances (bioactive polyphenols, dietary fiber, squalene, tocopherols), direct energy generation, bioenergy production (bioethanol, biohydrogen, biomethane, biodiesel), composting, biological detoxification, and use as animal feed [2,3,4].

The well recognized efficiency of insects to convert low quality organic matter into a biomass rich in high-quality protein and fat might represent a valuable solution in the handling of olive industry solid wastes. Yellow mealworm (*Tenebrio molitor* Linnaeus, 1758; Coleoptera: Tenebrionidae) larvae are one the most promising alternative protein and energy sources for food and feed [5,6]. Moreover, they show high plasticity in larval development time and survival rate, larval and pupal weight, and nutritional profile, depending on the feeding media [7,8,9,10,11,12,13]. Even though *T. molitor* is a cosmopolitan pest of stored grains, grain products and by-products, it can also consume many other agri-food by-products, bio-converting them for feed and food production in a circular economy view. A variety of feeding substrates have been studied for a decade: mixtures of dried potatoes and egg whites [7]; mixtures of spent and distillers’ grains, potato peelings, cookie and bread remains, beer yeast, and maize [9]; wheat and soybean flours added to bocaiuva (*Acrocomia aculeata* (Jacq.) Lodd) pulp flour [14]; mixtures of by-products from food manufacturing (beet molasses, potato peelings, spent grains, bread and cookies remains) [8]; watermelon rinds, eggshells, banana peels, and white bread [15]; mixtures of spent and distillers’ grains with wheat bran [12]; linseed added to wheat, oat, and corn flours [11]; mixtures of wheat bread and flours (wheat, oat, corn, chickpea) [10]; and by-products from maize production [16]. Even polystyrene foam [17] and fermented cattle dung mixed with conventional feed (wheat bran, corn flour, bean pulp) [18] have been investigated, but the bioconversion of olive pomace is still to be explored.

The present study aims to evaluate how feeding could affect growth performance and nutritional composition of yellow mealworm larvae fed substrates made up of organic wheat milling (low-grade flour) and olive processing by-products, in order to assess the coleopteran oil and proteins as potential food ingredients.

## 2. Materials and Methods 

### 2.1. Insect Feeding Media Preparation

Five different feeding media were tested: feed S1, 100% organic wheat flour; feed S2, 100% organic wheat middlings (both purchased from “Molino del Conero”, Osimo, Italy); and feeds S3, S4 and S5, organic wheat middlings enriched with 25%, 50%, and 75% of organic olive pomace (provided by “I tre filari” farm, Recanati, Italy), respectively. Olive pomace (moisture 60.33%) was processed in an electric homogenizer (Avent, Philips, Amsterdam, The Netherlands) before the feeding substrate preparation. Ingredients (wheat middlings and olive pomace) were mixed, homogenized and kept 24 h at 4 °C, before using.

### 2.2. Insect Rearing

*Tenebrio molitor* larvae were purchased from a local pet shop (PlanetFish & Co., Ancona, Italy). The mother colony was maintained at 28 ± 1 °C, 60 ± 5% RH, and 24 h dark photoperiod in plastic boxes (40 × 30 × 6 cm). Larvae were fed with organic wheat middlings and peeled organic carrots were used to supply moisture. Pupae were separated from the colony and allowed to complete development in smaller plastic boxes (20 × 15 × 6 cm). Newly emerged adults were placed in clean plastic trays (40 × 30 × 6 cm) lined with filter papers (Whatman, Dassel, Germany), and supplied with middlings and carrots. Eggs glued on the tray bottom were isolated and monitored until first instar hatched. The first instar larvae followed two different protocols:

#### 2.2.1. Insect Growth Performance Assessment

For each experimental feed, three replicates of 50 larvae each were placed in Petri dishes, together with 10 g of feed. Dishes were kept at 28 ± 1 °C, 60 ± 5% RH, and 24 h dark photoperiod. Fresh feed (10 g) and peeled carrots (2 g) were supplied weekly. We recorded the development time from the eclosion to the pupation of all surviving larvae, Moreover, the larval survival rate, the last larval instar weight, and the pupal weight were recorded.

#### 2.2.2. Insect Rearing for Chemical Analyses

For each experimental feed, three replicates of 4000 larvae, isolated with a very fine brush (20/0; Da Vinci-MICRONOVA, Nuremberg, Germany) and counted, were placed in plastic boxes (55 × 36 × 15 cm) and supplied with 0.25 g/larva of feed and 0.05 g/larva of carrots, until the occurrence of last instar larvae. Fresh feeds (0.125 g/larva) were added every two weeks, while organic carrots (0.05 g/larva) were supplied twice a week. Insects were reared in a climatic chamber at 28 ± 1 °C, 60 ± 5% RH and 24 h dark photoperiod. Last instar larvae were starved for 24 h before collecting, freeze-drying, grinding, and storing them under vacuum at −20 °C until analyses. Moreover, the residual products (mixture of feeding substrate residues, excreta, and exuviae) were stored under vacuum at −20 °C until analyses.

### 2.3. Proximate Composition

Proximate parameters (crude fat, crude protein, fiber, and total ash) of dried feeds and larvae were determined using the Association of Official Analytical Chemists methods [19]. Lipids were extracted by an automated Soxhlet apparatus (BÜCHI Labortechnik AG, Flawil, Switzerland) under inert gas (nitrogen), using petroleum ether as solvent. Protein amount was determined by using the Kjeldahl procedure and 6.25 as conversion coefficient. Nitrogen free extract (NFE) was calculated by difference from 100% dry matter (DM). Moisture content of larvae was evaluated by difference in weight before and after the freeze-drying process. Conversion factors reported in Regulation (EU) No 1169/2011, Annex XIV were used for the calculation of feed and larva energy values.

### 2.4. Fatty Acid Analysis

Fatty acid methyl esters (FAMEs) were prepared by acid-catalyzed transesterification of lipid extracts and analyzed by gas chromatography, according to the procedure and conditions described in Haddad et al. [20,21]. Fatty acid (FA) compositions (weight % of total FA) were calculated by the peak area normalization method.

### 2.5. Amino Acids Analysis

Protein hydrolysis was performed by treating feeding substrates and mealworm powders with 6 N HCl, at 110 °C for 24 h, under vacuum, in presence of 3 mM sarcosine as internal standard. For tryptophan determination, a basic hydrolysis was performed with 5 N NaOH, at 120 °C for 24 h. Details concerning derivatization of amino acids and HPLC separations were described in Roncolini et al. [22].

### 2.6. Anti-Trypsin and Anti-Chymotrypsin Analysis

Frozen larvae were homogenized in 50 mM TRIS-HCl pH 8.0, 50 mM NaCl, 1 mM tris(2-carboxyethyl) phosphine (TCEP). After centrifugation at 20,000 g for 15 minutes at 4 °C, supernatants were assayed for the anti-proteases activity before and after 5 min incubation at 100 °C. Assay mixtures (0.5 mL) consisted of 50 mM TRIS-HCl, pH 8.0 and 0.1 mg (4 Units) trypsin with 0.2 mM Nα-benzoyl-DL-arginine p-nitroanilide (BApNA) or 0.1 mg (4 Units) chymotrypsin with 0.1 mM N-succinyl-ala-ala-pro-phe p- nitroanilide (SAAPFpNA). Incubation was performed at 37 °C, and the absorbance was monitored at 410 nm. Proteases activity values were plotted against the amounts of supernatant, and from the slope, the amount of protease inhibited per mg of larvae was calculated.

### 2.7. Data Analysis

A one-way analysis of variance (ANOVA) was carried out to evaluate differences among feed compositions, among the chemical parameters of larvae fed on different feeds, and among development time, last larval instar weight, and pupal weight. The Tukey-Kramer’s Honest Significant Difference (HSD) test at the level of significance 0.05 was chosen for multiple means comparisons. The Kaplan–Meier estimator was used to estimate the survival function of larvae reared on different substrates. The Log-rank test at the level of significance 0.05 was used to check the null hypothesis (the survival curves of larvae grown on two or more substrates are equal). Variable reduction was achieved by PCA (Principal Component Analysis) on variance-covariance matrix, to assess the relationships among nutritional characteristics of edible larvae and their growing performance. Data auto scaling was used to optimally describe the orientation of scores and loadings. The software JMP Version 11.0.0 (SAS Institute Inc., Cary, NC, USA) was used to conduct all tests. 

## 3. Results and Discussion

### 3.1. Composition of Feeding Substrates 

Proximate compositions of the feeding substrates are reported in Table 1. As expected, lipid, protein, mineral, and fiber contents of middlings (S2) were significantly higher than wheat flour (S1), due to the presence of cereal grain parts other than endosperm (germ, aleurone layer). The olive pomace showed the following composition: 4.51% crude protein, 19.78% crude fat, 35.60% fiber, 31.43% NFE, 8.69% ash, on DM basis. Incorporation of increasing percentages of olive pomace (feeds S3–S5) resulted in a significant increase in moisture (from 8.14% to 53.16%), fiber (from 9.54% to 31.45% DM), and fat (from 5.73% to 7.44% DM), while crude protein percentage decreased from 16.84% to 11.76%, on DM basis. Feed S5 showed the highest moisture, fat, fiber, and ash levels, while feed S2 had the highest protein percentage and feed S1 the highest NFE percentage and energy value.

Table 2 reports the FA composition of total lipids extracted from feeding substrates. FA composition of control feeds (wheat flour, S1; middlings, S2) were in the range of previously published data [23]. Unsaturated fatty acids (UFAs) were the most abundant in all feeds, which accounted for 80%–83% of total FAs, because of oleic acid (91%–94% of total monounsaturated fatty acids, MUFAs) and linoleic acid (87%–93% of total polyunsaturated fatty acids, PUFAs) amounts. The inclusion of olive pomace increased the oleic acid relative percentage, from 19.26%–21.79% (S1, S2) to 59.98% (S5), and decreased the amount of linoleic acid, from 59.58%–60.43% (S1, S2) to 22.32% (S5), while changes of total saturated fatty acid (SFA) amounts were limited. A decrease of linolenic acid was also observed, as inclusion level of olive pomace increased, thus resulting in non-significant changes of the n-6/n-3 ratio of feeding substrates.

Feed proteins had a total amount of essential amino acids (EAA) ranging from 36.79% (S1) to 44.34% (S5) (Table 3). Middlings proteins had a higher EAA/NEAA (non-essential amino acids) ratio than wheat flour proteins, in agreement with the higher nutritional value of the middlings substrate. EAA/NEAA ratio was not affected by the substitution of the base feed (middlings) with increasing percentages of olive pomace. In all feeds, the most represented EAA were LEU, THR, MET and PHE, while the most abundant non-essential amino acids (NEAA) were GLU and PRO, which accounted for 26%–45% of total amino acids. 

### 3.2. Composition of Tenebrio Molitor Larvae

Proximate composition of *T. molitor* larvae reared on five different feeding substrates are listed in Table 4. Larvae mirrored the proximate data of their substrates. In fact, larvae L5 showed the highest moisture, fiber, and ash levels; larvae L2 showed the highest protein percentage; larvae L1 had the highest NFE percentage and energy value. The moisture percentage of larvae (60.50%–68.48%) was close to values reported by van Broekhoven et al. [9], for larvae fed with mixture of organic by-products, and higher than water contents reported by Ghaly et al [24], Siemianowska et al. [25], and Alves et al. [14], for mealworms fed whole wheat flour and brewer’s yeast, oat flakes, soybean and bocaiuva flours, respectively. Protein content observed in larvae receiving 100% middlings (L2) was higher than mealworms analyzed by Ravzanaadii et al. [26] (50.14% vs. 46.44% DM, respectively), which were only fed wheat bran. Larvae reared on 1:1 middlings/olive-pomace mixture (L4) also showed higher protein level (47.58% DM) than mealworms grown on flours mixture (wheat, soybean, bocaiuva pulp) (44.83%) and on mixtures of by-products from food manufacturing (watermelon, eggshells, banana peels), as reported by Alves et al. [14] and Tan et al. [15], respectively. Only experimental feeds made up of 95% whole wheat flour and 5% brewer’s yeast [24] allowed *T. molitor* larvae to reach a protein content as high as observed in the present study. Organic wheat flour (feed S1) was the best substrate to achieve the highest (40.10% DM) crude lipid content of mealworm, despite the higher fat content of feeds S2–S5. No significant differences in the fat content among larvae fed 100% middlings (L2) and pomace enriched middlings (L3–L5) were observed. Lipid levels agreed with values reported by Ravzanaadii et al. [26], Alves et al. [14], and Tan et al. [15]. No significant effect of the feeding substrate was detected for the fiber content of the five groups of larvae. Significant differences were registered for total soluble carbohydrates (NFE), ranging from 4.87% DM in mealworm fed S1 feed to 13.39% DM in larvae reared on 75% olive pomace substrate (L5). Tan et al. [15] found NFE values less than half in mealworms grown on feeds made up of common food wastes. Total mineral levels (ash) observed in the five mealworm groups were similar to those reported by Ghaly et al. [24], Siemianowska et al. [25], Alves et al. [14], and Tan et al. [15] for larvae grown on different feeding substrates.

The FA compositions of larvae lipids are reported in Table 5. UFAs (oleic and linoleic acids) were the most abundant in larvae lipids, while palmitic acid was the most representative SFA. FA composition of mealworm samples agreed with previously published data [5,9,10,11,12,14,22,25,26,27,28,29]. Significant differences were observed between the fat composition of larvae fed control substrates: mealworms grown on organic wheat flour (L1) exhibited higher oleic acid percentage and lower levels of PUFA (linoleic and α-linolenic acids) than larvae collected from middlings (L2), while lipids of larvae grown on feed S2 had the lowest percentages of oleic acid and the highest levels of PUFAs (linoleic and α-linolenic acids) and palmitic acid. The inclusion of pomace in the feed composition did not affect the FA composition of body lipids. Besides, no correlations were observed between the crude lipid content of larvae and of their feeding substrates, according to experimental data published by Francardi et al. [11], as a result of supplementation of feed with linseed. Dreassi et al. [10] also noticed that, despite the different fat levels of six cereal-based substrates, *T. molitor* larvae had the same fat percentage, whereas van Broekhoven et al. [9] found that dietary fat affected larval fat content.

Some authors highlighted the possibility of modifying the FA composition, and hence the nutritional quality, of the insect lipids during the breeding [10,11]. However, the FA composition of yellow mealworm samples collected from experimental substrates S1–S5 did not reflect the FA composition of feeds. van Broekhoven et al. [9] also observed that FA profile of mealworms grown on substrates composed of organic by-products of different origin (beer brewing, bread/cookie baking, potato processing, bioethanol production) did not follow the same trend of dietary FAs. Alves et al. [14] reported that the addition of 50% of bocaiuva flour to control feed (50% wheat flour, 50% soybean flour) did not significantly affect the FAs of the larvae lipids, and Oonincx et al. [8] highlighted that the fat composition of yellow mealworm seems to be fairly constant, in spite of n-6/n-3 PUFA ratio differences among feeds (various by-products derived from food manufacturing). This behavior might be attributed to the ability of yellow mealworm to de novo synthesize both linoleic and α-linolenic acids [11,30,31], which are essential for humans and other mammalians.

The amino acid composition of mealworm proteins is reported in Table 6. Larvae fed the five substrates showed a similar amino acid composition, with a total amount of EAA ranging from 48.71% to 53.69% and an EAA/NEAA ratio around 1.0. The most abundant EAA were THR (9.47%–11.14%), LEU (7.31%–8.49%), MET (7.62%–8.36%), and TYR (5.97%–8.40%), while the most represented NEAA were GLU (10.69%–11.38%), PRO (6.33%–10.7%7), ALA (7.89%–9.12%), and ASP (7.10%–8.11%). These values confirmed the well documented ability of insect proteins to meet the human requirement of essential amino acids [32] and indicated that larvae fed on all tested substrates are high-value protein sources, especially samples L3 and L4 which were characterized by the highest EAA/NEAA ratio (1.16).

Protease inhibitors, specifically trypsin and chymotrypsin inhibitors, represent one of the most relevant antinutritional factors in legume seeds, as they are resistant to digestion and inhibit the activity of key pancreatic enzymes in the gut, thereby reducing digestion and absorption of dietary proteins [33]. Table 7 reports on the presence of some anti-chymotrypsin activity in *T. molitor* larvae reared on the different substrates: higher values were observed in L2 and L4 samples, even if no significant differences (Tukey test, *p* < 0.05) were detected among groups. Furthermore, no inhibitory activity of trypsin was detected. However, the measured activities were about one order of magnitude lower than described in legume seeds [34]. Therefore, the presence of this anti-nutritional factor in *T. molitor* larvae did not represent a real safety problem. Notably, the inhibitory activity was resistant to heat treatment, as boiled extracts of larvae fully retained such activity (data not shown).

### 3.3. Insect Growth Performance

The availability of different nutrients affects important life history traits of insects, such as body weight, development time, and survival rate [35]. In holometabolous insects, all growth occurs before metamorphosis so that the final weight of the last instar larva determines the size and performance of the adult [36]. Moreover, pupal weight, which is strongly correlated with potential adult fecundity [37], was regularly incorporated into mass rearing systems as a measure of larval dietary quality. Survival rate and development time were also indicators of dietary quality [8].

The mean larval development time of experimental insects ranged between 98 (L2) and 133 days (L5), depending on feed (Table 8) and in accord with previously published data [8,9]. The shortest values were measured for larvae reared on control feed (100% wheat middlings, L2) and on feed having 25% of olive pomace (L3). Further increase of olive pomace percentage in larvae feed lengthened their development time. The last larval instar weight ranged from 0.070 to 0.131 g. It was significantly higher in larvae fed on control substrates (L1, L2) and on 25% pomace enriched feed (L3) than in substrates made up of 50%–75% of olive pomace (L4, L5). Final weights of larvae L1–L3 were similar to those of larvae fed on different substrates containing 10.25%–25.25% protein, 4%–14.92% fat, and 19.4%–61.17% carbohydrates [9,38]. Pupal weight ranged from 0.153 to 0.192 g depending on feed. It was significantly higher in larvae fed on S2 and S3 substrates than on the other rearing substrates. Larval survival rate ranged from 54% (L4) to 85% (L2). In particular, larvae fed on S1, S2 and S3 substrates showed significantly higher survival rates than larvae reared on highly pomace enriched media.

**Figure 1:**
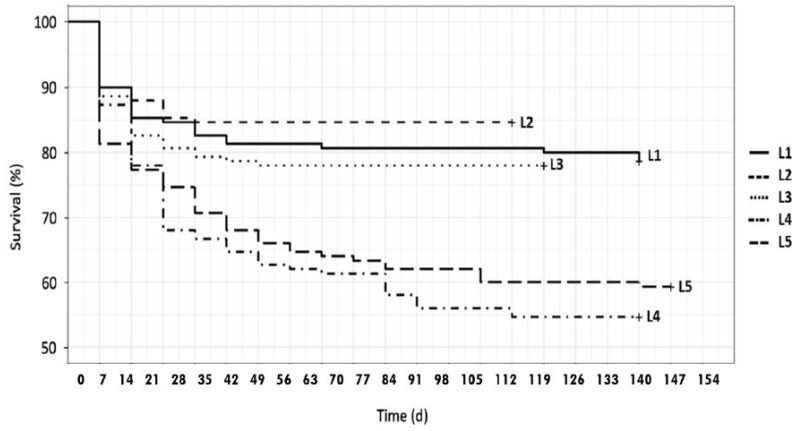
Survival rate of mealworm larvae reared on different feeding media, from eclosion to first pupa occurred. Survival rate was compared using Kaplan–Meier analysis and a pairwise comparison using Log-Rank test. p-values significant at *p* < 0.05. Larva identifiers (L1–L5) are as in Table 4.

Kaplan–Meier survival curves (Figure 1) for larvae fed on substrates S1–S3 were significantly different than feeds S4–S5 (S1–S4 *p* < 0.0001; S1–S5 *p* = 0.00072; S2–S4 *p* < 0.0001; S2–S5 *p* < 0.0001; S3–S4 *p* = 0.00017; S3–S5 *p* = 0.00211). Most of the larval mortality occurred within the first four weeks in all experiments, according to the behavior observed by Kim et al [39]. 

Mealworm larvae are able to self-select optimal nutrients from mixture of heterogeneous substrates in order to reach proper nutritional balance and guarantee best fitness [40]. Even if some dietary self-selection could not be excluded, mass balance of the *T. molitor* larvae reared on substrates S3 and S4 (Table S2) showed that the total fat intake was greater than the fat contribution of middlings moieties. The FA composition of lipids extracted from the residual products of larvae fed on S3 and S4 (Table S3) were much more similar to the FA composition of larval feeding substrates (Table 2) than the FA profile of olive pomace. On the other hand, the residual product of larvae fed on S5 showed a lipid composition very close to olive pomace.

Protein and carbohydrate are the two major nutrients that affect larval growth performance and survival rate in insects [41]. The carbohydrates/protein ratio affects development time and larval growth of *T. molitor* [40,42]. Insects can synthetize lipids out of different dietary components such as protein and carbohydrates [43,44]. Mealworm larvae have been reared with success on a variety of feeding substrates with a fat range from 4% to 29% and a moisture up to 22% [9,10,14,45]. In our experiment, mealworm larvae performed better in feeds (S2, S3) having more than 50% carbohydrates, more than 15% proteins and a fat range from 5.73% to 6.18% (Table 1). Furthermore, the presence of moisture in the diet is very important for the performance of *T. molitor* [46,47,48], although it inhabit water-deficient environments too. In our experiments, L4 and L5 fed on diets containing more than 22% of moisture showed the worsts grow performance and survival rate. It could also be hypothesized that the presence of high level of polyphenolic compounds affected the growth performance of mealworm larvae fed on S4 and S5. In facts, phenols are able to form complexes with proteins, thus affecting protein digestibility [49], amino acid absorption and assimilation and resulting in a decreased larval growth and survival rate [50,51]. 

### 3.4. Nutritional Quality and Growth Performance of Mealworm Larvae

**Figure 2:**
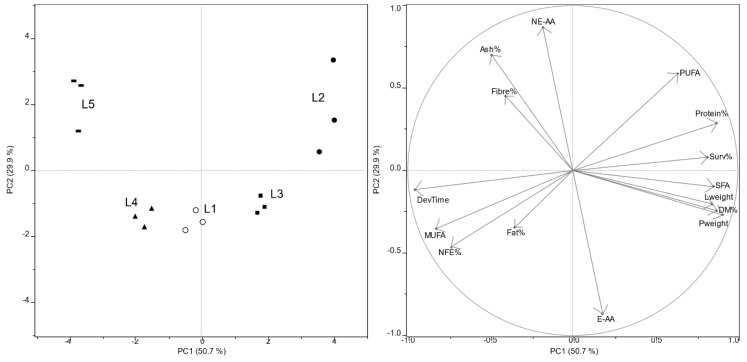
Left: PCA scores plot of larvae fed on five different substrates. Larva identifiers (L1–L5) are as in Table 4. Right: PCA loadings plot of variables (nutritional and growth performance parameters). Abbreviations not elsewhere described are: DevTime, development time; Surv%, survival percentage; Lweight, last larval instar weight; Pweight, pupal weight.

Experimental data were explored by PCA to evaluate the structure of variables (main nutritional parameters and growth performance of mealworm larvae) and objects (larvae samples collected from three replicates of five feeding substrates) (Figure 2). Control substrates (organic wheat flour and middlings) showed a different behavior, as they were fully separated on both PC1 and PC2: larvae fed middlings (L2) grew better (higher larval and pupal weights, higher survival rate, lower development time) with a higher DM yield and protein content than larvae fed flour (L1). Larvae reared on the 25% of olive pomace lied on the lower-right quarter of the scores plot, which represented the best compromise between growth performance and nutritional properties. In fact, survival %, larval and pupal weight, together with DM% and protein content had the highest positive loadings on PC1, while PC2 was mainly affected by NEAA (high positive loading) and EAA (high negative loading). Further increase of olive pomace percentage in middlings-based feeds drove larvae (L4, L5) to negative loadings on PC1, thus worsening growth performance, reducing DM yield and protein content of larvae.

## 4. Conclusions

The present paper confirms the ability of *T. molitor* larvae to convert food industry by-products into a biomass that could be exploited for both food and feed. It might be a helpful solution in the managing of solid waste produced by olive oil industry, which represents a severe environmental challenge in the Mediterranean area. A feeding substrate made up of 25% olive pomace and 75% wheat middlings (S3) appeared to be the best compromise between growth performance (larval and pupal weights, survival rate, development time) and nutritional properties (amount and quality of proteins and fat) of mealworm larvae. In fact, larvae fed S3 feed showed the highest DM yield, protein content, and EAA/NEAA ratio. Fat content and FA composition of larvae reared on S3 substrate were not significantly different than those detected on larvae fed more pomace-enriched feeds (S4, S5). Further trials are needed to improve the lipid composition of mealworm larvae, whose n-6/n-3 ratio is quite far from optimal for human nutrition. L3 larvae also had the lowest level of antinutritional factors (chymotrypsin inhibitors) among the middlings based substrates.

## Figures and Tables

**Table 1:** Proximate composition (mean ± SD, *n* = 3) of feeding substrates^1^.

.	S1	S2	S3	S4	S5
Moisture (%)	9.66 ± 0.03 ^d^	8.14 ± 0.14 ^d^	21.96 ± 0.17 ^c^	37.42 ± 0.48 ^b^	53.16 ± 0.78 ^a^
Protein (% DM)	13.19 ± 0.01 ^c^	16.84 ± 0.18 ^a^	15.42 ± 0.15 ^b^	14.89 ± 0.30 ^b^	11.76 ± 0.10 ^d^
Fat (% DM)	1.20 ± 0.03 ^e^	5.73 ± 0.03 ^d^	6.18 ± 0.05 ^c^	6.99 ± 0.14 ^b^	7.44 ± 0.06 ^a^
Fiber (% DM)	0.58 ± 0.03 ^e^	9.54 ± 0.25 ^d^	16.77 ± 0.21 ^c^	21.58 ± 0.13 ^b^	31.45 ± 0.34 ^a^
NFE (% DM)	84.22 ± 0.02 ^a^	62.84 ± 0.71 ^b^	56.89 ± 0.45 ^c^	51.67 ± 0.67 ^d^	44.11 ± 0.35 ^e^
Ash (% DM)	0.82 ± 0.03 ^b^	5.06 ± 0.25 ^a^	4.76 ± 0.05 ^a^	4.88 ± 0.11 ^a^	5.25 ± 0.16 ^a^
Energy content (kcal/100 g DM)	402 ± 0 ^a^	389 ± 1 ^b^	378 ± 0 ^c^	372 ± 0 ^d^	353 ± 0 ^e^

^1^ Feeding substrates were (% *w*/*w*): S1 organic wheat flour (100); S2 middlings (100); S3 middlings (75) + olive pomace (25); S4 middlings (50) + olive pomace (50); S5 middlings (25) + olive pomace (75). Values in a row with different letters are significantly different (Tukey test, *p* < 0.05).

**Table 2:** Fatty acid composition (mean ± SD, *n* = 3) of feeding substrates ^1^.

FA [*w*/*w* %]	S1	S2	S3	S4	S5
C8:0 ^2^	0.02 ± 0.01	0.01 ± 0.00	0.01 ± 0.01	0.02 ± 0.01	0.01 ± 0.01
C10:0	0.01 ± 0.01	0.01 ± 0.00	0.01 ± 0.01	0.01 ± 0.01	0.01 ± 0.00
C12:0	0.03 ± 0.06	0.02 ± 0.01	0.03 ± 0.01	0.02 ± 0.01	0.02 ± 0.01
C13:0	tr	tr	tr	tr	tr
C14:0	0.15 ± 0.01 ^a^	0.12 ± 0.01 ^b^	0.12 ± 0.01 ^b^	0.10 ± 0.01 ^b^	0.07 ± 0.01 ^c^
C14:1∆9	tr	tr	tr	tr	tr
C15:0	0.12 ± 0.01 ^a^	0.11 ± 0.01 ^a^	0.11 ± 0.01 ^ab^	0.08 ± 0.01 ^b^	0.04 ± 0.01 ^c^
C16:0	17.67 ± 0.55 ^a^	15.80 ± 0.65 ^bc^	17.23 ± 0.46 ^ab^	16.99 ± 0.65 ^abc^	15.64 ± 0.60 ^c^
C16:1	0.18 ± 0.02 ^d^	0.19 ± 0.02 ^d^	0.47 ± 0.01 ^c^	0.69 ± 0.01 ^b^	0.83 ± 0.01 ^a^
C17:0	0.10 ± 0.01 ^a^	0.07 ± 0.01 ^b^	0.07 ± 0.01 ^b^	0.07 ± 0.01 ^b^	0.06 ± 0.00 ^b^
C17:1∆10	0.09 ± 0.01 ^ab^	0.07 ± 0.00 ^c^	0.09 ± 0.01 ^ab^	0.08 ± 0.01 ^bc^	0.11 ± 0.01 ^a^
C18:0	1.47 ± 0.04 ^a^	1.05 ± 0.06 ^b^	1.38 ± 0.04 ^a^	1.59 ± 0.09 ^a^	1.00 ± 0.23 ^b^
C18:1∆9	17.61 ± 0.84 ^e^	20.02 ± 0.76 ^d^	33.91 ± 0.77 ^c^	45.33 ± 0.66 ^b^	56.54 ± 0.46 ^a^
C18:1Δ11	0.80 ± 0.03 ^d^	0.81 ± 0.02 ^d^	1.39 ± 0.02 ^c^	1.80 ± 0.02 ^b^	2.04 ± 0.07 ^a^
C18:2 n-6	56.49 ± 0.68 ^a^	55.15 ± 0.22 ^b^	40.73 ± 0.65 ^c^	29.40 ± 0.33 ^d^	20.80 ± 0.27 ^e^
C20:0	0.12 ± 0.02 ^d^	0.14 ± 0.01 ^cd^	0.17 ± 0.01 ^c^	0.25 ± 0.02 ^b^	0.32 ± 0.01 ^a^
C18:3 n-3	3.84 ± 0.16 ^b^	4.34 ± 0.19 ^a^	2.95 ± 0.06 ^c^	2.18 ± 0.12 ^d^	1.45 ± 0.03 ^e^
C20:1∆11	0.58 ± 0.03 ^b^	0.70 ± 0.02 ^a^	0.62 ± 0.01 ^b^	0.52 ± 0.01 ^c^	0.46 ± 0.03 ^d^
C20:2∆11,14	0.09 ± 0.01 ^ab^	0.09 ± 0.02 ^b^	0.11 ± 0.01 ^a^	0.10 ± 0.01 ^ab^	0.07 ± 0.01 ^b^
SFA	19.68 ± 0.51 ^a^	17.33 ± 0.65 ^b^	19.14 ± 0.42 ^a^	19.13 ± 0.65 ^a^	17.16 ± 0.55 ^b^
MUFA	19.26 ± 0.84 ^e^	21.79 ± 0.78 ^d^	36.49 ± 0.76 ^c^	48.42 ± 0.69 ^b^	59.98 ± 0.48 ^a^
PUFA	60.43 ± 0.75 ^a^	59.58 ± 0.07 ^a^	43.80 ± 0.59 ^b^	31.68 ± 0.20 ^c^	22.32 ± 0.23 ^d^
n-6/n-3	14.74 ± 0.57 ^a^	12.74 ± 0.62 ^b^	13.85 ± 0.48 ^ab^	13.59 ± 0.89 ^ab^	14.43 ± 0.48 ^a^

^1.^ Feeding substrate identifiers are as in Table 1. Values in a row with different letters are significantly different (Tukey test, *p* < 0.05). ^2^ Cm:n ∆x, m = number of carbon atoms; n, number of double bonds; x, position of double bonds; SFA, saturated fatty acids; MUFA, monounsaturated fatty acids; PUFA, polyunsaturated fatty acids; tr, trace (<0.01%).

**Table 3:** Amino acid percentage composition (mean ± SD, *n* = 3) of feeding substrates ^1^.

	S1	S2	S3	S4	S5
HIS	2.30 ± 0.33	3.31 ± 0.25	3.06 ± 0.57	2.64 ± 0.98	2.78 ± 0.48
THR	5.60 ± 0.97 ^b^	9.53 ± 0.07 ^a^	8.94 ± 0.63 ^a^	9.83 ± 0.89 ^a^	9.42 ± 0.03 ^a^
TYR	2.13 ± 0.06	2.65 ± 0.23	2.41 ± 0.45	2.22 ± 0.10	2.41 ± 0.41
VAL	1.21 ± 0.01 ^a^	0.99 ± 0.04 ^b^	0.95 ± 0.04 ^bc^	0.76 ± 0.09 ^c^	0.87 ± 0.03 ^bc^
MET	5.53 ± 0.20 ^b^	6.75 ± 0.23 ^a^	6.48 ± 0.10 ^a^	6.59 ± 0.24 ^a^	6.66 ± 0.29 ^a^
PHE	5.60 ± 0.02	4.89 ± 0.72	4.61 ± 0.10	4.73 ± 0.10	4.86 ± 0.16
ILE	3.81 ± 0.29	3.60 ± 0.25	3.63 ± 0.42	3.42 ± 0.41	3.89 ± 0.26
LEU	7.47 ± 0.38	7.71 ± 1.04	7.63 ± 0.12	7.64 ± 0.06	7.86 ± 0.32
LYS	2.33 ± 0.46	2.66 ± 0.65	3.16 ± 1.17	5.06 ± 0.96	4.82 ± 1.92
TRP	0.81 ± 0.02 ^ab^	1.24 ± 0.27 ^a^	0.86 ± 0.28 ^ab^	0.44 ± 0.06 ^b^	0.77 ± 0.19 ^ab^
Total EAA	36.79 ± 1.52 ^b^	43.31 ± 1.81 ^a^	41.73 ± 1.69 ^ab^	43.33 ± 1.08 ^a^	44.34 ± 1.14 ^a^
ASP	4.70 ± 0.63 ^b^	8.40 ± 0.01 ^a^	7.99 ± 0.97 ^a^	7.29 ± 0.10 ^a^	7.92 ± 0.37 ^a^
GLU	33.69 ± 1.27 ^a^	20.39 ± 2.43 ^b^	22.25 ± 0.73 ^b^	21.15 ± 0.55 ^b^	20.82 ± 0.04 ^b^
SER	4.49 ± 0.08 ^b^	5.78 ± 0.07 ^a^	5.57 ± 0.42 ^a^	5.47 ± 0.10 ^a^	5.52 ± 0.03 ^a^
GLY	1.86 ± 0.06 ^b^	2.61 ± 0.10 ^a^	2.58 ± 0.28 ^a^	2.52 ± 0.05 ^a^	2.61 ± 0.10 ^a^
ARG	4.16 ± 0.13 ^b^	7.65 ± 0.18 ^a^	7.34 ± 0.94 ^a^	7.39 ± 0.46 ^a^	6.73 ± 0.85 ^a^
ALA	3.54 ± 0.18 ^b^	5.92 ± 0.15 ^a^	5.63 ± 0.46 ^a^	5.53 ± 0.17 ^a^	5.65 ± 0.29 ^a^
PRO	10.76 ± 0.45 ^a^	5.95 ± 0.79 ^b^	6.90 ± 0.64 ^b^	7.34 ± 0.50 ^b^	6.42 ± 0.48 ^b^
Total NEAA	63.21 ± 1.52 ^a^	56.69 ± 1.81 ^b^	58.27 ± 1.69 ^ab^	56.67 ± 1.08 ^b^	55.66 ± 1.14 ^b^
EAA/NEAA	0.58 ^b^	0.77 ^a^	0.72 ^ab^	0.76 ^a^	0.80 ^a^

^1^ Feeding substrate identifiers are as in Table 1. EAA, essential amino acids; NEAA, non-essential amino acids. Values in a row with different letters are significantly different (Tukey test, *p* < 0.05).

**Table 4:** Proximate composition (mean ± SD, *n* = 3) of *T. molitor* larvae ^1^ reared on different feeding substrates.

	L1	L2	L3	L4	L5
Moisture (%)	64.73 ± 0.90 ^b^	60.50 ± 0.67 ^d^	61.95 ± 1.26 ^cd^	63.32 ± 0.67 ^bc^	68.48 ± 0.76 ^a^
Protein (% DM)	37.78 ± 0.74 ^b^	50.14 ± 2.42 ^a^	47.58 ± 1.59 ^a^	39.39 ± 1.33 ^b^	38.05 ± 0.94 ^b^
Fat (% DM)	40.10 ± 1.31 ^a^	34.04 ± 4.00 ^b^	32.14 ± 1.79 ^b^	35.32 ± 0.60 ^ab^	36.06 ± 1.90 ^ab^
Fiber (% DM)	5.97 ± 0.31 ^a^	7.06 ± 0.64 ^a^	8.34 ± 0.29 ^a^	7.97 ± 1.27 ^a^	10.18 ± 3.45 ^a^
NFE (% DM)	12.68 ± 0.64 ^ab^	4.87 ± 0.96 ^c^	8.08 ± 0.54 ^bc^	13.39 ± 3.04 ^a^	11.14 ± 2.14 ^ab^
Ash (% DM)	3.48 ± 0.10 ^b^	3.90 ± 0.30 ^b^	3.86 ± 0.16 ^b^	3.92 ± 0.27 ^b^	4.57 ± 0.18 ^a^
Energy content (kcal/100 g DM)	575 ± 7 ^a^	540 ± 22 ^ab^	529 ± 8 ^b^	545 ± 1 ^ab^	542 ± 15 ^ab^

^1^ Larvae were reared on organic wheat flour (L1), middlings (L2), middlings/olive pomace 75:25 *w*/*w* (L3), middlings/olive pomace 50:50 *w*/*w* (L4), middlings/olive pomace 25:75 *w*/*w* (L5). Values in a row with different letters are significantly different (Tukey test, *p* < 0.05).

**Table 5:** Fatty acid composition (mean ± SD, *n* = 3) of *T. molitor* larvae ^1^ reared on different feeding substrates.

FA (*w*/*w* %)	L1	L2	L3	L4	L5
C8:0 ^2^	0.02 ± 0.01	0.03 ± 0.02	0.01 ± 0.00	0.01 ± 0.00	0.02 ± 0.01
C10:0	0.04 ± 0.01 ^a^	0.03 ± 0.01 ^b^	0.02 ± 0.00 ^b^	0.02 ± 0.00 ^b^	0.02 ± 0.00 ^b^
C12:0	0.44 ± 0.06 ^a^	0.46 ± 0.07 ^a^	0.35 ± 0.02 ^a^	0.21 ± 0.02 ^b^	0.18 ± 0.02 ^b^
C13:0	0.03 ± 0.01 ^b^	0.07 ± 0.01 ^a^	0.05 ± 0.01 ^b^	0.03 ± 0.00 ^b^	0.03 ± 0.00 ^b^
C14:0	4.79 ± 0.58 ^a^	4.28 ± 0.25 ^ab^	3.73 ± 0.17 ^b^	2.61 ± 0.13 ^c^	2.35 ± 0.09 ^c^
C14:1∆9	0.02 ± 0.01 ^a^	0.02 ± 0.00 ^a^	0.01 ± 0.00 ^b^	0.01 ± 0.00 ^b^	0.01 ± 0.01 ^b^
C15:0	0.06 ± 0.01 ^b^	0.13 ± 0.02 ^a^	0.11 ± 0.02 ^a^	0.10 ± 0.00 ^a^	0.11 ± 0.01 ^a^
C16:0	16.72 ± 0.77 ^ab^	18.16 ± 0.58 ^a^	16.10 ± 0.85 ^bc^	15.94 ± 0.56 ^bc^	14.93 ± 0.26 ^c^
C16:1	3.00 ± 0.18 ^a^	1.71 ± 0.13 ^b^	1.35 ± 0.06 ^c^	1.74 ± 0.12 ^b^	1.52 ± 0.03 ^bc^
C17:0	0.07 ± 0.01 ^b^	0.12 ± 0.03 ^a^	0.11 ± 0.01 ^a^	0.12 ± 0.01 ^a^	0.14 ± 0.01 ^a^
C17:1∆10	0.11 ± 0.01 ^b^	0.09 ± 0.01 ^b^	0.11 ± 0.02 ^b^	0.17 ± 0.02 ^a^	0.17 ± 0.01 ^a^
C18:0	2.58 ± 0.18 ^b^	2.73 ± 0.02 ^ab^	3.01 ± 0.17 ^a^	2.54 ± 0.07 ^b^	2.51 ± 0.04 ^b^
C18:1∆9	52.63 ± 1.23 ^b^	45.06 ± 1.92 ^c^	55.98 ± 0.75 ^a^	56.58 ± 0.22 ^a^	58.04 ± 0.22 ^a^
C18:1Δ11	0.11 ± 0.01 ^d^	0.30 ± 0.05 ^c^	0.54 ± 0.06 ^b^	0.57 ± 0.03 ^b^	0.69 ± 0.01 ^a^
C18:2 n-6	18.81 ± 0.22 ^b^	25.37 ± 2.41 ^a^	17.71 ± 0.96 ^b^	18.53 ± 0.47 ^b^	18.50 ± 0.16 ^b^
C20:0	0.11 ± 0.01 ^b^	0.11 ± 0.01 ^b^	0.19 ± 0.03 ^a^	0.13 ± 0.01 ^b^	0.09 ± 0.00 ^b^
C18:3 n-3	0.30 ± 0.00 ^c^	1.00 ± 0.13 ^a^	0.42 ± 0.01 ^bc^	0.51 ± 0.02 ^b^	0.47 ± 0.02 ^b^
C20:1∆11	0.06 ± 0.02 ^b^	0.13 ± 0.03 ^a^	0.10 ± 0.01 ^ab^	0.09 ± 0.01 ^ab^	0.12 ± 0.00 ^a^
C20:2∆11,14	0.08 ± 0.02 ^b^	0.15 ± 0.03 ^a^	0.11 ± 0.03 ^ab^	0.10 ± 0.01 ^ab^	0.11 ± 0.01 ^ab^
SFA	24.85 ± 1.48 ^a^	26.11 ± 0.82 ^a^	23.67 ± 0.96 ^ab^	21.72 ± 0.74 ^bc^	20.37 ± 0.33 ^c^
MUFA	55.92 ± 1.26 ^b^	47.30 ± 2.00 ^c^	58.09 ± 0.74 ^ab^	59.16 ± 0.26 ^a^	60.56 ± 0.19 ^a^
PUFA	19.18 ± 0.25 ^b^	26.52 ± 2.57 ^a^	18.24 ± 0.98 ^b^	19.14 ± 0.49 ^b^	19.08 ± 0.16 ^b^
n-6/n-3 ratio	62.94 ± 0.82 ^a^	25.52 ± 1.00 ^d^	42.77 ± 2.16 ^b^	36.77 ± 0.22 ^c^	39.37 ± 1.76 ^bc^

^1.^ Larva identifiers are as in Table 4. Values in a row with different letters are significantly different (Tukey test, *p* < 0.05). ^2^ Cm:n ∆x, m = number of carbon atoms; n, number of double bonds; x, position of double bonds; SFA, saturated fatty acids; MUFA, monounsaturated fatty acids; PUFA, polyunsaturated fatty acids; tr, trace (< 0.01%).

**Table 6:** Amino acid percentage composition (mean ± SD, *n* = 3) of *T. molitor* larvae ^1^ reared on different feeding substrates.

	L1	L2	L3	L4	L5
HIS	3.21 ± 0.29 ^bc^	1.56 ± 0.73 ^d^	4.99 ± 0.39 ^a^	4.74 ± 0.45 ^ab^	2.53 ± 0.90 ^cd^
THR	10.09 ± 0.53	11.14 ± 0.52	10.02 ± 0.23	9.47 ± 0.11	9.62 ± 1.40
TYR	8.40 ± 0.13 ^a^	5.97 ± 0.20 ^b^	6.99 ± 0.24 ^ab^	7.60 ± 0.94 ^a^	6.93 ± 0.86 ^ab^
VAL	0.99 ± 0.08	0.97 ± 0.05	1.04 ± 0.06	0.91 ± 0.08	0.97 ± 0.09
MET	8.22 ± 0.52	7.62 ± 0.71	8.36 ± 0.35	8.12 ± 0.20	7.63 ± 0.42
PHE	3.46 ± 0.08 ^b^	3.52 ± 0.09 ^b^	3.79 ± 0.10 ^ab^	3.90 ± 0.17 ^a^	3.47 ± 0.21 ^b^
ILE	4.67 ± 0.42 ^a^	3.86 ± 0.20 ^b^	4.31 ± 0.17 ^ab^	4.61 ± 0.09 ^a^	4.43 ± 0.30 ^ab^
LEU	7.31 ± 0.29 ^b^	8.49 ± 0.45 ^a^	7.79 ± 0.36 ^ab^	7.89 ± 0.13 ^ab^	7.40 ± 0.32 ^b^
LYS	4.94 ± 0.48	6.29 ± 0.46	5.99 ± 0.82	6.02 ± 0.36	5.09 ± 1.04
TRP	0.58 ± 0.04 ^ab^	0.72 ± 0.04 ^a^	0.41 ± 0.04 ^c^	0.43 ± 0.02 ^bc^	0.65 ± 0.11 ^a^
Total EAA	51.86 ± 1.00 ^ab^	50.15 ± 1.10 ^bc^	53.68 ± 0.28 ^a^	53.69 ± 0.26 ^a^	48.71 ± 0.92 ^c^
ASP	7.53 ± 0.44	7.34 ± 0.48	7.10 ± 0.32	7.64 ± 0.90	8.11 ± 0.42
GLU	10.89 ± 0.35 ^ab^	11.38 ± 0.23 ^a^	11.10 ± 0.26 ^ab^	11.32 ± 0.16 ^ab^	10.69 ± 0.19 ^b^
SER	5.03 ± 0.05 ^ab^	4.88 ± 0.19 ^ab^	5.18 ± 0.09 ^a^	4.81 ± 0.12 ^b^	4.77 ± 0.13 ^b^
GLY	2.58 ± 0.12 ^a^	1.74 ± 0.23 ^b^	2.70 ± 0.05 ^a^	2.84 ± 0.32 ^a^	2.64 ± 0.39 ^a^
ARG	5.68 ± 0.04 ^ab^	6.06 ± 0.23 ^a^	5.48 ± 0.16 ^ab^	5.31 ± 0.42 ^b^	5.80 ± 0.20 ^ab^
ALA	9.12 ± 0.31 ^a^	8.34 ± 0.27 ^ab^	8.42 ± 0.08 ^ab^	7.89 ± 0.54 ^b^	8.51 ± 0.22 ^ab^
PRO	7.31 ± 0.28 ^b^	10.12 ± 1.10 ^a^	6.33 ± 0.18 ^b^	6.49 ± 0.25 ^b^	10.77 ± 1.32 ^a^
Total NEAA	48.14 ± 1.00 ^bc^	49.85 ± 1.10 ^ab^	46.32 ± 0.28 ^c^	46.31 ± 0.26 ^c^	51.29 ± 0.92 ^a^
EAA/NEAA	1.08 ^ab^	1.01 ^bc^	1.16 ^a^	1.16 ^a^	0.95 ^c^

^1^ Larva identifiers are as in Table 4. EAA, essential amino acids; NEAA, non-essential amino acids. Values in a row with different letters are significantly different (Tukey test, *p* < 0.05).

**Table 7:** Chymotrypsin inhibitory activity (µg of trypsin inhibited/g of larva) of *T. molitor* larvae ^1^ reared on different feeding substrates.

L1	L2	L3	L4	L5
1.22 ± 0.32	2.31± 0.48	1.69 ± 0.43	2.39 ± 0.69	2.14 ± 0.55

^1^ Larva identifiers are as in Table 4.

**Table 8:** Growth performance and survival rate (mean ± SD, *n* = 150) of *T. molitor* larvae ^1^ reared on different feeding substrates.

	L1	L2	L3	L4	L5
Development time (days)	137 ± 5 ^ab^	109 ± 5 ^c^	112 ± 0 ^bc^	140 ± 7 ^a^	147 ± 7 ^a^
Survival (%)	79 ± 4.0 ^a^	85 ± 3.1 ^a^	78 ± 2.0 ^a^	54 ± 10.6 ^b^	58 ± 4.0 ^b^
Last larval instar weight (g)	0.113 ± 0.003 ^a^	0.128 ± 0.004 ^a^	0.131 ± 0.004 ^a^	0.081 ± 0.003 ^b^	0.070 ± 0.003 ^b^
Pupal weight (g)	0.176 ± 0.030 ^b^	0.190 ± 0.047 ^a^	0.192 ± 0.042 ^a^	0.171 ± 0.037 ^b^	0.153 ± 0.030 ^c^

^1^ Larva identifiers are as in Table 4. Values in a row with different letters are significantly different (Tukey test, *p* < 0.05).

## Supplementary Materials

The following are available online at https://www.mdpi.com/2304-8158/9/3/317/s1, Table S1: Proximate composition and energy value (mean ± SD, n = 3) of the residual product (mixture of feeding substrate residues, excreta, and exuviae) from the T. molitor larvae’s growth process; Table S2: Mass balance (mean values, n = 3) of the T. molitor larvae reared on two-component feeding substrates (S3–S5); Table S3: Fatty acid composition (mean ± SD, n = 3) of the residual products (mixture of feeding substrate residues, excreta, and exuviae) of the T. molitor larvae and of olive pomace used as ingredients in feeds S3–S5; Table S4: Microbiological characterization of insect feeding substrates [29,52,53,54,55,56,57,58,59,60].
