# Supplementary material for: Addition of Olive Pomace to Feeding Substrate Affects Growth Performance and Nutritional Value of Mealworm (Tenebrio Molitor L.) Larvae
Source: Foods. 2020 Mar 10;9(3):317. doi: 10.3390/foods9030317 (PMC7143744; doi:10.3390/foods9030317)
Supplement: Supplementary file 1 [file foods-09-00317-s001.zip › foods-724740 supplementary materials/foods-724740-supplementary.docx]

Microbiological Characterization of Insect Feeding Substrates

1. Introduction

The European Food Safety Authority (EFSA) recently reviewed all available information on the safety of insects for use in feed and food [52]. According to the EFSA recommendations, the authors contributed to the study of insect microbiota and investigated the bacterial biota characterizing a pilot production of *Tenebrio molitor* larvae reared on wheat milling by-products [53]. Data concerning the microbiological quality of substrates described in the main paper are provided here, with the aim of contributing to the safety assessment of the final product (mealworm larvae).

2. Materials and Methods

Twenty-five grams of each diet (S1–S5) were suspended in 225 mL of sterilized peptone water (1 g L^-1^, Oxoid, Basingstoke, UK) and subjected to 5 min of homogenization at 230 rpm in a Stomacher 400 Circulator apparatus (VWR International PBI, Milan, Italy). Suspensions were then 10-fold diluted in peptone water and subjected to microbial enumeration of total mesophilic aerobes, lactic acid bacteria, *Enterobacteriaceae*, and spore forming bacteria in appropriate growth media, as already described in Osimani et al. [53]. Eumycetes were counted as previously reported in Osimani et al. [29]. The presence of *Listeria monocytogenes* and *Salmonella* spp. were assessed according to the AFNOR BIO 12/11-03/04 and AFNOR BIO 12/16-09/05 standard methods, respectively. Microbiological analyses were performed in triplicate.

3. Results and discussion

Viable counts of the experimental diets are reported in Table S1.

**Table S4.** Microbiological characterization of the different feeding media (diets ^1^). The results are expressed as mean value (log cfu g^-1^) ± standard deviation.

| **Sample** | **Total Mesophilic Aerobes** | **Lactic Acid Bacteria** | ***Enterobacteriaceae*** | **Spore Forming Bacteria** | **Eumycetes** |
| --- | --- | --- | --- | --- | --- |
| S1 | 2.74 ± 0.02 | 1.39 ± 0.12 | 3.96 ± 0.07 | 2.81 ± 0.04 | 1.45 ± 0.21 |
| S2 | 5.19 ± 0.02 | 4.98 ± 0.10 | 4.24 ± 0.16 | 3.46 ± 0.20 | 3.44 ± 0.11 |
| S3 | 4.64 ± 0.20 | 3.84 ± 0.05 | 3.87 ± 0.08 | 2.49 ± 0.08 | 3.89 ± 0.06 |
| S4 | 5.11 ± 0.02 | 5.15 ± 0.05 | 2.59 ± 0.18 | 1.15 ± 1.63 | 5.28 ± 0.00 |
| S5 | 6.24 ± 0.02 | 6.33 ± 0.01 | n.d. | 1.30 ± 0.43 | 6.46 ± 0.04 |
| OP | 6.08 ± 0.02 | 5.95 ± 0.08 | 1.00 ± 0.00 | 0.65 ± 0.92 | 6.33 ± 0.07 |

^1^ S1: 100% organic wheat flour; S2: 100% organic wheat middlings; S3: 25% organic wheat middlings / 75% organic olive pomace; S3: 50% organic wheat middlings / 50% organic olive pomace; S3: 75% organic wheat middlings / 25% organic olive pomace; OP: 100% organic olive pomace. n.d., not detectable.

Counts of total mesophilic aerobes ranged between 2.74 and 6.24 log cfu g^-1^, whereas average values for lactic acid bacteria were comprised between 1.39 and 6.33 log cfu g*-1*. *Enterobacteriaceae* ranged between 1.00 and 3.96 log cfu g^-1^; no counts were evidenced in S5. Spore forming bacteria were between 0.65 and 2.81 log cfu g^-1^, while Eumycetes were between 1.45 and 6.46 log cfu g^-1^. *L. monocytogenes* and *Salmonella* spp. were not detected in any samples analyzed.

The counts of total mesophilic aerobes and spore forming bacteria were comparable with those obtained by Osimani et al. [53] in the same rearing substrate, whereas lactic acid bacteria and *Enterobacteriaceae* were notably higher than those previously counted by the same authors. It is known that *Enterobacteriaceae* are suitable hygiene indicators, hence their massive presence in the rearing substrate can affect the final microbiota of mealworms. Total mesophilic aerobes and spore forming bacteria of organic wheat flour were in the range reported by Alfonzo et al. [54], whereas *Enterobacteriaceae* were higher than those reported by Burns et al. [55]. Spore forming bacteria in organic wheat flour were in the range reported by Valerio et al. [56]. Regarding eumycetes counted in the two cereal-based feeding substrates, it is noteworthy that such microbial group naturally occurs in cereal matrices. The presence of *Enterobacteriaceae* in olive pomace has already been reported by Pepi et al. [57] whereas Ertuğrul et al. [58] demonstrated the presence of spore forming bacteria (*Bacillus* spp.) in olive mill wastewater. Olive wastes could have high Eumycetes and lactic acid bacteria loads, that are typically found on olive surface and oil production environment [59, 60], thus explaining the high counts of these two microbial groups in this substrate. Finally, the mixed feeds reflected the microbial loads detected in the starting raw materials.

References

29. Osimani, A.; Garofalo, C.; Milanović, V.; Taccari, M.; Cardinali, F.; Aquilanti, L.; Pasquini, A.; Mozzon, M.; Raffaelli, N.; Ruschioni, S.; Riolo, P.; Isidoro, N.; Clementi, F. Insight into the proximate composition and microbial diversity of edible insects marketed in the European Union. *Eur Food Res Technol*. **2017**, 243, 1157–1171.

1. EFSA Scientific Committee. Risk profile related to production and consumption of insects as food and feed. *EFSA Journal* **2015**, 13, 4257.
2. Osimani, A.; Milanović, V.; Cardinali, F.; Garofalo, C.; Clementi, F.; Pasquini, M.; Riolo, P.; Ruschioni, S.; Isidoro, N.; Loreto, N.; Franciosi, E.; Tuohy, K.; Petruzzelli, A.; Foglini, M.; Gabucci, C.; Tonucci, F.; Aquilanti, L. The bacterial biota of laboratory-reared edible mealworms (*Tenebrio molitor* L.): From feed to frass. *Int J Food Microbiol* **2018**, 272, 49–60.
3. Alfonzo, A.; Miceli, C.; Nasca, A.; Franciosi, E.; Ventimiglia, G.; Di Gerlando, R.; Tuohy, K.; Francesca, N.; Moschetti, G.; Settanni, L. Monitoring of wheat lactic acid bacteria from the field until the first step of dough fermentation. *Food Microbiol* **2017**, 62, 256–269.
4. Burns, A.M.; Lawlor, P.G.; Gardiner, G.E.; McCabe, E.M.; Walsh, D.; Mohammed, M.; Grant, J.; Duffy, G. *Salmonella* occurrence and *Enterobacteriaceae* counts in pig feed ingredients and compound feed from feed mills in Ireland. *Prev Vet Med* **2015**, 121, 231–239.
5. Valerio, F.; De Bellis, P.; Di Biase, M.; Lonigro, S.L.; Giussani, B.; Visconti, A.; Lavermicocca, P.; Sisto, A. Diversity of spore-forming bacteria and identification of *Bacillus amyloliquefaciens* as a species frequently associated with the ropy spoilage of bread. *Int J Food Microbiol* **2012**, 156, 278–285.
6. Pepi, M.; Lampariello, L.R.; Altieri, R.; Esposito, A.; Perra, G.; Renzi, M.; Lobianco, A.; Feola, A.; Gasperini, S.; Focardi, S.E. Tannic acid degradation by bacterial strains *Serratia* spp. and *Pantoea* sp. isolated from olive mill waste mixtures. *Int Biodeter Biodegrad* **2010**, 64, 73–80.
7. Ertuğrul, S.; Dönmez, G.; Takaç, S. Isolation of lipase producing *Bacillus* sp. from olive mill wastewater and improving its enzyme activity. *J Hazard Mater* **2007**, 149, 720–724.
8. Ciafardini, G.; Zullo, B.A. Virgin olive oil yeasts: A review. *Food Microbiol* **2018**, 70, 245-253.
9. De Angelis, M.; Campanella, D.; Cosmai, L.; Summo, C.; Rizzello, C.G.; Caponio, F. Microbiota and metabolome of un-started and started Greek-type fermentation of Bella di Cerignola table olives. *Food Microbiol* **2015**, 52, 18-30.

| 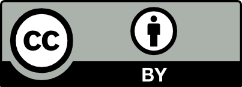 | © 2020 by the authors. Submitted for possible open access publication under the terms and conditions of the Creative Commons Attribution (CC BY) license (http://creativecommons.org/licenses/by/4.0/). |
| --- | --- |
