# Supplementary material for: Addition of Olive Pomace to Feeding Substrate Affects Growth Performance and Nutritional Value of Mealworm (Tenebrio Molitor L.) Larvae
Source: Foods. 2020 Mar 10;9(3):317. doi: 10.3390/foods9030317 (PMC7143744; doi:10.3390/foods9030317)
Supplement: Supplementary file 1 [file foods-09-00317-s001.zip › foods-724740 supplementary materials/s001.docx]

**Table S1.** Proximate composition and energy value (mean ± SD, *n* = 3) of the residual product (mixture of feeding substrate residues, excreta, and exuviae) from the *T. molitor* larvae’s growth process.

|  | **S1-res** | **S2-res** | **S3-res** | **S4-res** | **S5-res** |
| --- | --- | --- | --- | --- | --- |
| Moisture (%) | 12.56 ± 0.31 ^a^ | 9.69 ± 0.44 ^b^ | 10.86 ± 0.14 ^ab^ | 11.60 ± 0.43 ^a^ | 11.80 ± 1.25 ^a^ |
| Protein (% DM) | 15.61 ± 0.40 ^b^ | 21.41 ± 2.00 ^a^ | 17.54 ± 1.05 ^ab^ | 12.69 ± 3.36 ^bc^ | 9.88 ± 1.76 ^c^ |
| Fat (% DM) | 0.62 ± 0.04 ^d^ | 2.74 ± 0.48 ^c^ | 2.75 ± 0.29 ^c^ | 5.53 ± 1.21 ^b^ | 10.84 ± 0.56 ^a^ |
| Fiber (% DM) | 1.41 ± 0.19 ^c^ | 19.01 ± 1.30 ^b^ | 28.25 ± 3.85 ^ab^ | 38.49 ± 3.12 ^a^ | 32.18 ± 8.41 ^a^ |
| NFE (% DM) | 81.20 ± 0.65 ^a^ | 47.76 ± 2.94 ^b^ | 42.48 ± 3.18 ^bc^ | 37.58 ± 2.61 ^c^ | 41.18 ± 6.76 ^bc^ |
| Ash (% DM) | 1.16 ± 0.05 ^c^ | 9.07 ± 0.39 ^a^ | 8.99 ± 0.67 ^a^ | 5.70 ± 0.71 ^b^ | 5.92 ± 0.69 ^b^ |
| Energy content (kcal/100 g DM) | 396 ± 0 ^a^ | 339 ± 6 ^c^ | 321 ± 8 ^c^ | 328 ± 10 ^c^ | 366 ± 16 ^b^ |

Original feeding substrates were (% *w*/*w*): S1 organic wheat flour (100); S2 middlings (100); S3 middlings (75) + olive pomace (25); S4 middlings (50) + olive pomace (50); S5 middlings (25) + olive pomace (75). Values in a row with different letters are significantly different (Tukey test, *p* < 0.05).
