# Supplementary material for: Addition of Olive Pomace to Feeding Substrate Affects Growth Performance and Nutritional Value of Mealworm (Tenebrio Molitor L.) Larvae
Source: Foods. 2020 Mar 10;9(3):317. doi: 10.3390/foods9030317 (PMC7143744; doi:10.3390/foods9030317)
Supplement: Supplementary file 1 [file foods-09-00317-s001.zip › foods-724740 supplementary materials/s002.docx]

**Table S2.** Mass balance (mean values, *n* = 3) of the *T. molitor* larvae reared on two-component feeding substrates (S3-S5).

|  | **S3** | **S4** | **S5** |
| --- | --- | --- | --- |
| Total diet (g) | 3000 | 4167 | 4833 |
| Middlings (g) (A) | 2250 | 2084 | 1208 |
| Water (g) | 183 | 170 | 98 |
| Fat (g) | 118 | 110 | 64 |
| Fiber (g) | 197 | 183 | 106 |
| Protein (g) | 348 | 322 | 187 |
| Ash (g) | 105 | 97 | 56 |
| NFE (g) | 1299 | 1203 | 697 |
| Olive Pomace (g) (B) | 750 | 2084 | 3625 |
| Water (g) | 452 | 1257 | 2187 |
| Fat (g) | 59 | 163 | 284 |
| Fiber (g) | 106 | 294 | 512 |
| Protein (g) | 13 | 37 | 65 |
| Ash (g) | 26 | 72 | 125 |
| NFE (g) | 94 | 260 | 452 |
| Residual (g) (C) | 1993 | 2574 | 3072 |
| Water (g) | 216 | 299 | 362 |
| Fat (g) | 49 | 126 | 294 |
| Fiber (g) | 502 | 876 | 872 |
| Protein (g) | 312 | 289 | 268 |
| Ash (g) | 160 | 130 | 160 |
| NFE (g) | 755 | 855 | 1116 |
| Total intake (A + B - C) | 1007 | 1593 | 1761 |
| Water (g) | 419 | 1128 | 1923 |
| Fat (g) | 128 | 147 | 54 |
| Fiber (g) | -199 | -399 | -254 |
| Protein (g) | 50 | 71 | -16 |
| Ash (g) | -29 | 39 | 21 |
| NFE (g) | 638 | 607 | 34 |

Original feeding substrates were (% *w*/*w*): S3 middlings (75) + olive pomace (25); S4 middlings (50) + olive pomace (50); S5 middlings (25) + olive pomace (75).
