# Supplementary material for: Addition of Olive Pomace to Feeding Substrate Affects Growth Performance and Nutritional Value of Mealworm (Tenebrio Molitor L.) Larvae
Source: Foods. 2020 Mar 10;9(3):317. doi: 10.3390/foods9030317 (PMC7143744; doi:10.3390/foods9030317)
Supplement: Supplementary file 1 [file foods-09-00317-s001.zip › foods-724740 supplementary materials/s003.docx]

**Table S3.** Fatty acid composition (mean ± SD, *n* = 3) of the residual products (mixture of feeding substrate residues, excreta, and exuviae) of the *T. molitor* larvae and of olive pomace used as ingredients in feeds S3–S5.

| **FA [*w*/*w* %]** | **S1-res** | **S2-res** | **S3-res** | **S4-res** | **S5-res** | **Olive pomace** |
| --- | --- | --- | --- | --- | --- | --- |
| C8:0 | 0.04 ± 0.01 | 0.04 ± 0.02 | 0.01 ± 0.01 | 0.01 ± 0.01 | 0.01 ± 0.00 | - |
| C10:0 | 0.05 ± 0.02 | 0.04 ± 0.01 | 0.01 ± 0.01 | 0.01 ± 0.00 | 0.01 ± 0,00 | - |
| C12:0 | 0.45 ± 0.35 | 0.04 ± 0.00 | 0.02 ± 0.01 | 0.01 ± 0.00 | 0.05 ± 0.02 | - |
| C13:0 | 0.01 ± 0.01 | tr | tr | tr | tr | - |
| C14:0 | 0.95 ± 0.11 | 0.28 ± 0.02 | 0.16 ± 0.01 | 0.09 ± 0.03 | 0.06 ± 0.00 | - |
| C14:1∆9 | 0.01 ± 0.01 | tr | 0.01 ± 0.01 | tr | tr | - |
| C15:0 | 0.12 ± 0.01 | 0.12 ± 0.01 | 0.04 ± 0.00 | 0.02 ± 0.01 | 0.02 ± 0.00 | - |
| C16:0 | 13.67 ± 0.25 | 15.02 ± 0.87 | 11.70 ± 0.41 | 12.52 ± 0.64 | 13.15 ± 0.43 | 11.93 |
| C16:1 | 0.60 ± 0.21 | 0.26 ± 0.02 | 0.81 ± 0.02 | 0.95 ± 0.08 | 0.98 ± 0.06 | 0.71 |
| C17:0 | 0.17 ± 0.01 | 0.09 ± 0.02 | 0.06 ± 0.01 | 0.07 ± 0.01 | 0.07 ± 0.01 | 0.10 |
| C17:1∆10 | 0.37 ± 0.13 | 0.09 ± 0.02 | 0.10 ± 0.03 | 0.12 ± 0.03 | 0.11 ± 0.01 | 0.08 |
| C18:0 | 3.49 ± 0.04 | 1.05 ± 0.63 | 1.39 ± 1.33 | 2.19 ± 0.15 | 2.15 ± 0.06 | 2.32 |
| C18:1∆9 | 25.60 ± 1.86 | 23.16 ± 0.57 | 40.76 ± 0.29 | 49.96 ± 0.74 | 69.10 ± 0.14 | 72.62 |
| C18:1Δ11 | 0.73 ± 0.08 | 0.79 ± 0.11 | 1.36 ± 0.48 | 2.25 ± 0.03 | 2.25 ± 0.05 | 1.01 |
| C18:2 n-6 | 50.32 ± 1.17 | 53.37 ± 0.74 | 40.37 ± 0.93 | 28.91 ± 1.09 | 10.46 ± 0.29 | 9.77 |
| C20:0 | 0.27 ± 0.02 | 0.30 ± 0.05 | 0.45 ± 0.05 | 0.43 ± 0.11 | 0.33 ± 0.02 | 0.42 |
| C18:3 n-3 | 2.57 ± 0.15 | 4.15 ± 0.11 | 2.05 ± 0.06 | 1.84 ± 0.03 | 0.74 ± 0.03 | 0.73 |
| C20:1∆11 | 0.51 ± 0.02 | 1.07 ± 0.11 | 0.64 ± 0.06 | 0.42 ± 0.04 | 0.34 ± 0.03 | 0.31 |
| C20:2∆11,14 | 0.13 ± 0.01 | 0.14 ± 0.01 | 0.11 ± 0.01 | 0.17 ± 0.01 | 0.17 ± 0.02 | - |

Original feeding substrates were (% *w*/*w*): S1 organic wheat flour (100); S2 middlings (100); S3 middlings (75) + olive pomace (25); S4 middlings (50) + olive pomace (50); S5 middlings (25) + olive pomace (75). ^.^Cm:n ∆x, m = number of carbon atoms; n, number of double bonds; x, position of double bonds; tr, trace (<0.01%).
